# Utilization of the PICO framework to improve searching PubMed for clinical questions

**DOI:** 10.1186/1472-6947-7-16

**Published:** 2007-06-15

**Authors:** Connie Schardt, Martha B Adams, Thomas Owens, Sheri Keitz, Paul Fontelo

**Affiliations:** 1 Medical Center Library, Duke University, DUMC Box 3702, Durham, North Carolina, 27710, USA; 2 Department of Medicine, Duke University Medical Center, Box 3228, Durham, North Carolina, 27710, USA; 3 Department of Medicine, Duke University Medical Center, Box 3675, Durham, North Carolina, 27710, USA; 4 Department of Medicine (111), Miami VAMC, 1201 NW 16th St., Miami, Florida 33125, USA; 5 National Library of Medicine, 8600 Rockville Pike, Bethesda, Maryland, 20894, USA

## Abstract

**Background:**

Supporting 21^st ^century health care and the practice of evidence-based medicine (EBM) requires ubiquitous access to clinical information and to knowledge-based resources to answer clinical questions. Many questions go unanswered, however, due to lack of skills in formulating questions, crafting effective search strategies, and accessing databases to identify best levels of evidence.

**Methods:**

This randomized trial was designed as a pilot study to measure the relevancy of search results using three different interfaces for the PubMed search system. Two of the search interfaces utilized a specific framework called PICO, which was designed to focus clinical questions and to prompt for publication type or type of question asked. The third interface was the standard PubMed interface readily available on the Web. Study subjects were recruited from interns and residents on an inpatient general medicine rotation at an academic medical center in the US. Thirty-one subjects were randomized to one of the three interfaces, given 3 clinical questions, and asked to search PubMed for a set of relevant articles that would provide an answer for each question. The success of the search results was determined by a precision score, which compared the number of relevant or gold standard articles retrieved in a result set to the total number of articles retrieved in that set.

**Results:**

Participants using the PICO templates (Protocol A or Protocol B) had higher precision scores for each question than the participants who used Protocol C, the standard PubMed Web interface. (Question 1: A = 35%, B = 28%, C = 20%; Question 2: A = 5%, B = 6%, C = 4%; Question 3: A = 1%, B = 0%, C = 0%) 95% confidence intervals were calculated for the precision for each question using a lower boundary of zero. However, the 95% confidence limits were overlapping, suggesting no statistical difference between the groups.

**Conclusion:**

Due to the small number of searches for each arm, this pilot study could not demonstrate a statistically significant difference between the search protocols. However there was a trend towards higher precision that needs to be investigated in a larger study to determine if PICO can improve the relevancy of search results.

## Background

Practicing evidence-based medicine (EBM) requires integration of clinical experience, the best available research evidence, and the values and preferences of the patient into the clinical decision-making process [1]. The steps in practicing EBM are centered on the patient and involve asking well-focused questions, searching for the best available evidence, appraising that evidence for validity, and then applying the results to the care of the patient. Supporting 21^st ^century health care and the practice of EBM requires ubiquitous access to clinical information and to knowledge-based resources. Clinicians and educators currently utilize a variety of resources and interfaces to search the biomedical literature to answer clinical questions. PubMed is a service of the U.S. National Library of Medicine (NLM) that provides access to over 16 million citations from MEDLINE and other life science journals dating back to the 1950s [2]. Since 1997, PubMed has been freely available to physicians, researchers, and the public. The information obtained from literature searches in PubMed can have a significant impact on patient care and clinical outcomes. Crowley et al reported on a study of 625 clinical questions asked by residents during an in-hospital general medicine rotation. Seventy-seven percent of the answers to these questions came from MEDLINE and the information from the articles changed patient management 47% of the time [3]. Klein et al. showed that conducting a MEDLINE search early in the hospitalization of a patient could significantly lower costs, charges, and lengths of stay [4]. Westbrook et al reported that the use of an online information retrieval system improved the quality of clinicians' answers to clinical questions by 21% [5]. The literature also reports that many clinical questions go unanswered due to difficulties formulating a relevant question [6], forgetting the question [7], lack of access to information resources, and lack of skills in searching [8].

Formulating a well-focused question is the first and arguably the most important step in the EBM process. Without a well-focused question, it can be very difficult and time consuming to identify appropriate resources and search for relevant evidence. Practitioners of EBM often use a specialized framework, called PICO, to form the question and facilitate the literature search. PICO stands for Patient problem, Intervention, Comparison, and Outcome. [9] The PICO framework can be expanded to PICOTT, adding information about the Type of question being asked (therapy, diagnosis, prognosis, harm, etc.) and the best Type of study design for that particular question. Using this framework helps the clinician articulate the important parts of the clinical question most applicable to the patient and facilitates the searching process by identifying the key concepts for an effective search strategy. [10,11]

PubMed includes a feature called Clinical Queries, which helps identify citations with appropriate study design by linking the type of question (therapy, diagnosis, etiology and prognosis) to a stored search strategy that retrieves the appropriate research methodology. The Clinical Queries are based on extensive research by the Hedges Study Team at McMaster University [12] and have been shown to improve searching results. Combining the PICO framework with the PubMed Clinical Queries has the potential to improve the efficiency of literature searching. Bergus et al reported that questions with at least a defined intervention (I) and outcome (O) were more likely to be answered than questions with one or none of these parameters [13]. The objectives of this pilot study were to measure the relevancy or precision of PubMed search results when using a PICO search framework, to test feasibility of the study design and to identify possible trends.

## Methods

**Figure 1:**
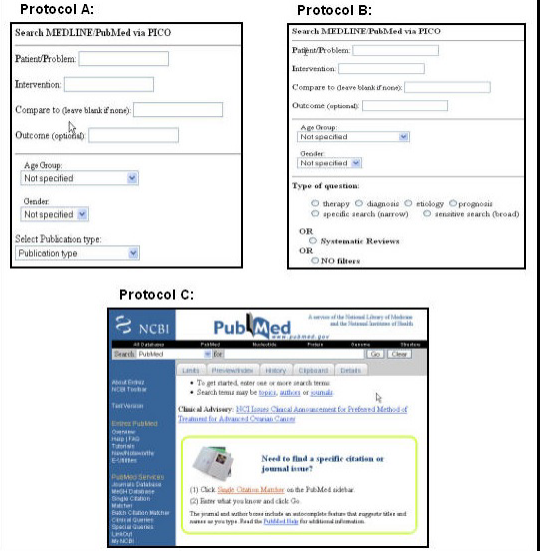
Three interfaces used in searching PubMed.

This randomized trial was designed as a pilot study to measure the relevancy of search results using three different interfaces for the PubMed search system. Each participant was randomly assigned to one of 3 interfaces for searching PubMed (Figure 1). Protocol A, [14] used a PubMed/PICO template designed for use on a wireless handheld device. The PubMed/PICO template prompted the searcher for the PICO elements of the question (patient problem, intervention, comparison and outcome), as well as patient age group and gender. There was also an option to select a publication type. The publication types listed were clinical trial, randomized controlled trial, meta-analysis, review or practice guideline. If no publication type was selected, the search default was to include all five study designs. The PubMed/PICO template with these publication options was designed to favor questions of therapy, the most common type of question asked by clinicians [3]. Protocol B, [15] used a PubMed/PICO/Clinical Queries template that prompted for the PICO and allowed the search to be filtered by type of question and scope of strategy (narrow or broad) or by systematic review. Protocol B incorporated the Clinical Queries filters and allowed the searcher to consider a broader range of question types. Because templates for Protocols A and B were designed for handheld devices, participants assigned to these two protocols were given a handheld device of their choice (either a Palm™ Tungsten C or a HP iPAC Pocket PC™) to use during the study. The Web browser home page for the wireless handheld study devices was preconfigured to connect to the appropriate study interface. Protocol C, PubMed, [2] used the standard web-based PubMed system on a PC workstation. Protocol C did not include a PICO template for formulating the search strategy, but the participants had access to Clinical Queries if they chose to use them.

Study subjects were recruited from interns and residents on an inpatient general medicine rotation at an academic medical center in the US. Thirty-one subjects were each given three clinical questions and asked to search PubMed for a set of relevant articles that would provide an answer to the questions. The three study questions were taken from a database of actual clinical questions formulated by residents during general medicine rotations between 2001 and 2002 [3]. Two of the questions (Q2 and Q3) were related to treatment or therapy, the most common type of question asked and one question (Q1) was related to prognosis. (Table 2.) While the PICO framework was developed specifically for therapy questions [16], the prognosis question was included because PICO is being taught for all types of questions. Protocol assignments and the 3 clinical questions were placed in a concealed envelope, and participants were asked to select one envelope from a group of identical envelopes. Participants were instructed to search each clinical question, as many times as needed, in order to retrieve a final set of articles that would provide the relevant information needed to make a clinical decision in each case.

**Table 1:** Baseline characteristics of study participants

	**Protocol A N = 10**	**Protocol B N = 10**	**Protocol C N = 10**
Interns	5	5	4
Residents	5	4	5
Other	0	0	1
Previous EBM training	5	9	8
Previous searching training	10	9	9
**How often do you search MEDLINE?**			
Daily	3	3	3
Weekly	6	3	5
Monthly or less	1	3	2

**Table 2:** Precision of search results

	**PICO A**	**95% CI**	**PICO B**	**95% CI**	**PubMed**	**95% CI**
**Q1**	35%	16.40% – 53.60%	28%	15.84% – 40.16%	20%	10.70% -29.30%
**Q2**	5%	1.42% – 8.58%	6%	2.42% – 9.58%-	4%	1.50% – 6.50%
**Q3**	1%	-0.43% – 2.43%	1%	-0.14% – 2.14%	0%	0.00%

Q1: Does BMI affect mortality in obese patients undergoing a CABG?

Q2: Is survival prolonged if I place a PEG or feeding tube in my patient with dementia?

Q3: Is an ACE inhibitor alone better than a diuretic alone for reducing hypertension in African American patients?

The success of the search was measured by comparing the number of relevant citations retrieved to the total number of article retrieved in the final set. The research team identified the relevant articles for each clinical question. The criteria for an article being included as relevant was that it addressed the specific clinical question, including patient, intervention and outcome, and that it was of the best study methodology based on the type of question. For example, a therapy question needed to be answered by a randomized controlled trial, systematic review, or meta-analysis, while a prognosis question required a prospective cohort study. Two researchers conducted PubMed searches for each question. These two and a third researcher selected the relevant articles from the pooled results of the searches. A fourth researcher also reviewed the results and reconciled any disagreement among the other reviewers.

Participants using the handheld devices wrote down their IP address, the time, and the number of citations in the set that best addressed the clinical question. This information was matched with data collected by the project server at NLM and was used to verify the final result set. Participants using the PC workstation were asked to save their final set results to the study account in *MyNCBI*. Screen captures, a means of saving the image of the search, were made of their complete search history to back up their saved strategies. For all participants, the search terms, date and time of the search, and the Unique Identifiers for the citations retrieved were collected by the system transactions logs and stored on the NLM project server or in *MyNCBI*. There were no time constraints on any of the participants. The DUMC Institutional Review Board approved the study method and all participants signed an informed consent form.

## Results

The primary outcome measurement was the precision of the final result set selected to answer the clinical question. Precision was defined as the ratio of relevant citations retrieved to the total number of citations retrieved in the set. The higher the precision the more efficient the search, as more of what is retrieved is also relevant. The measurement of precision is appropriate in the clinical setting, where it is often desirable to find a few good articles, as opposed to a research setting, where the measurement of recall, finding all possible relevant articles, is more important. We also examined the number of terms and filters used in the search strategy and user satisfaction with the search interface.

Thirty-one residents completed the study. Ten residents were randomized to Protocol A; 10 residents were randomized to Protocol B; and 11 residents were randomized to Protocol C. The results from one participant in Protocol C were discarded because the saved search strategies were corrupted and not useable. All three groups had similar clinical experience and searching experience, although the participants in Protocol A had less training in EBM (Table 1). Participants using the PICO templates (Protocol A or Protocol B) had higher precision scores for each question than the participants who used the standard PubMed system in Protocol C. (Question 1: A = 35%, B = 28%, C = 20%; Question 2: A = 5%, B = 6%, C = 4%; Question 3: A = 1%, B = 0%, C = 0%) 95% confidence intervals were calculated for the precision for each question using a lower boundary of zero. The 95% confidence limits were overlapping, suggesting no statistical difference between the groups. Although there were no statistical differences between the groups, there may be a trend toward improved precision with the PICO search screens. (Table 2)

In addition to quantitative comparisons, this pilot study provided an opportunity for more open-ended insight into how practitioners search. Some searches within each question (Question 1 = 3/30, Question 2 = 9/30, and Question 3 = 27/30) did not retrieve any relevant citations. A qualitative assessment of search strategies that produced no acceptable results revealed three common types of errors: ambiguous mapping of subject headings (MeSH); selecting the wrong publication type; and limiting search queries to just words in the title. The error that accounted for the largest number of non-productive searches was related to the MEDLINE indexing structure or MeSH (Medical Subject Headings). In question three, most searchers used the phrase "*African Americans*", which maps to the MeSH "*African Americans*" and retrieves 29077 citations [searched 4/18/07]. However, the common word "*blacks*" maps to the broader MeSH term "*African continental ancestry group*" and retrieves 48195 citations [searched 4/18/07]. Most of the relevant citations used either the word "*blacks*" or were indexed to the broader MeSH term "*African continental ancestry group*." The second most common error was related to selecting the wrong study design or Clinical Query for the type of question being searched. This was a common problem for Protocol A, which only listed therapy study designs. The third error affected Protocol C and involved limiting search terms to the title field. While searchers often select their articles based on relevant words in the title, a problem arises when more than one word or phrase is appropriate to the topic. For example, a "peg" is also called a "percutaneous endoscopic gastrostomy tube" or "feeding tube." Limiting the search to the word "peg" in the title eliminates mapping to appropriate subject headings (MeSH) such as *intubation*, *gastrointestinal *and therefore may exclude relevant articles on the topic that use alternative terminology. Understanding these errors can help in teaching effective searching and in developing better search systems.

Perceptions of ease of use and time spent searching were approximately the same across all three protocols, as were the numbers of terms used in each search, regardless of protocol used. However, of the 30 searches performed using Protocol B (PubMed/PICO/Clinical Queries), 25 (83%) actually incorporated the Clinical Queries into the strategy, as opposed to only 2 (7%) of the searches using Protocol C (PubMed/Web). Both Protocol A and B facilitated the use of publication types and the Clinical Queries by prompting the searcher to consider these elements in the strategy. While these elements were also available in Protocol C, they are either behind the Limits tab or listed in the PubMed Services menu.

## Discussion

Effective searching requires a series of steps to lead the practitioner from the clinical question or bedside to informed decision making. In March of 2005, over 68,000,000 searches were conducted in PubMed, from seven million unique IP addresses. [17] This study attempted to show that targeted interfaces structured to help clinicians search PubMed using the PICO format could affect the relevancy of the results, which ultimately could improve patient care. While this study was intended to look only at the actual search process using the intermediate measure of precision, examining the impact this can have on clinical care requires further research. Even though the standard PubMed search screen provides access to the Clinical Queries function, searchers in Protocol C used it infrequently. Placing the Clinical Queries option directly on the search screen, as in Protocol B, increased the likelihood of their use. This study has several limitations. First, as this study was designed as a pilot for a larger study, the sample size was small and may not have been adequately powered to show a significant difference. Second, we provided the subjects with well-focused questions, which might have been unrealistic in their simplicity and transfer to the PICO template. In the clinical setting the work of generating questions from clinical situations and translating them into the PICO format may be much more difficult. The success of the search may be much more dependent on asking a good question than on the search framework. Third, we recruited our subjects from a busy general medicine rotation, where EBM is taught and residents are expected to search the literature to address clinical questions. These residents may be more skilled in database searching than at other institutions and therefore generalizing the results to a less experienced population may not be appropriate. Finally, searching PubMed from a handheld may have resulted in some frustration with the searching process, especially if participants were not familiar with the specific devices. Although all residents within the Internal Medicine Residency Program had been given PDAs, none had previously used the device to search PubMed. While this study was not designed to address the technical issues of using handheld devices for searching PubMed, we did test the effectiveness of the search template, which NLM had designed specifically for use on the PDA. Using a handheld device and a PICO template to search the biomedical literature can address some of the barriers of time and access to resources for answering clinical questions.

## Conclusion

Searches performed on a PICO-formatted screen retrieved a higher percentage of relevant citations than searches performed on the standard PubMed search interface. However, due to the small number of searches for each arm, this pilot study could not demonstrate a statistically significant difference between the search protocols. There was a suggestion of a trend towards higher precision that needs to be investigated in a larger study to determine if PICO can improve the relevancy of search results.

## Competing interests

The author(s) declare that they have no competing interests.

## Authors' contributions

CS and TO participated in the study design, carried out the study, analyzed the results, and contributed to the writing of the manuscript. MA participated in the study design, carried out the study, and contributed to the writing of the manuscript. SK participated in the study design, analyzed the results, and contributed to the writing of the manuscript. All authors read and approved the final manuscript. FP conceived of the study, participated in the study design, developed the PICO templates, and contributed to the writing of the manuscript. All authors read and approved the final manuscript.

## Pre-publication history

The pre-publication history for this paper can be accessed here:


